# Neonatal growth with fish oil or mixed oil lipid emulsions in intestinal failure associated liver disease

**DOI:** 10.1016/j.intf.2026.100381

**Published:** 2026-06-11

**Authors:** Emily A. Gutzwiller, Katie A. Huff

**Affiliations:** a Indiana University School of Medicine, Indianapolis, IN, USA; b Nationwide Children’s Hospital, Department of Pediatrics, Columbus, OH, USA; c Indiana University School of Medicine, Division of Neonatal-Perinatal Medicine, Indianapolis, IN, USA; d Cincinnati Children’s Hospital Medical Center, Division of Neonatology, Cincinnati, OH, USA; e University of Cincinnati College of Medicine, Department of Pediatrics, Cincinnati, OH, USA

**Keywords:** infant, newborn, intestinal diseases, cholestasis, parenteral nutrition, body size

## Abstract

**Background:**

Treatment strategies for intestinal failure associated liver disease (IFALD) include use of fish-oil lipid emulsion (FO-ILE) or mixed-oil emulsion containing soy, medium chain triglyceride, olive, and fish oils (SO,MCT,OO,FO-ILE). FO-ILE is limited to 1 g/kg/day, either limiting overall calorie provision or shifting calories toward carbohydrate predominance. The objective of this project was to compare the growth of neonates with IFALD receiving either SO,MCT,OO,FO-ILE or FO-ILE.

**Material and methods:**

A retrospective chart review was conducted of neonates with IFALD receiving SO,MCT,OO,FO-ILE or FO-ILE. Growth metrics, nutritional data, and lab values were analyzed using the Mann-Whitney or t-test with a p-value < 0.05 used for significance.

**Results:**

We included 46 patients (22 SO,MCT,OO,FO-ILE; 24 FO-ILE). Both groups had similar weight gain (SO,MCT,OO,FO-ILE 24.6 vs FO-ILE 31.6 g/day; p = 0.121), although the FO-ILE patients had increased weight z-scores over time (SO,MCT,OO,FO-ILE: −0.11 vs FO-ILE: 0.09 p = 0.032). The FO-ILE group received fewer calories (SO,MCT,OO,FO-ILE:112 vs FO-ILE:100.7 kcal/kg/day; p = 0.004), less lipid (SO,MCT,OO,FO-ILE: 2.1 vs FO-ILE 1.0 g/kg/day; p < 0.001), and more carbohydrate (SO,MCT,OO,FO-ILE: 9.6 vs FO-ILE: 12.8 mg/kg/min; p < 0.001).

**Conclusion:**

Neonates receiving FO-ILE had similar weight gain to those receiving SO,MCT,OO,FO-ILE while receiving fewer overall calories and parenteral lipid. The FO-ILE group did have increased weight z-score over time. Further investigation is needed to understand the influence of these differences on additional short-term and long-term outcomes.

## Introduction

Intestinal failure (IF) is a clinical disorder characterized by inadequate intestinal function to sustain fluid, energy, electrolyte, or nutrient balance, with a need for parenteral nutrition (PN) [1], [2]. Neonates with IF require PN for survival; however, prolonged PN exposure is associated with significant complications, including intestinal failure-associated liver disease (IFALD). IFALD is a form of cholestatic liver disease, most often defined as a direct or conjugated bilirubin level ≥ 2 mg/dL in the absence of another identifiable cause, following a minimum of two weeks of PN exposure [2]. IFALD affects about 50% of infants receiving prolonged PN and can be a severe, potentially life-limiting condition [3].

The pathogenesis of IFALD is likely multifactorial, with intravenous lipid emulsions (ILE), particularly soybean oil-based intravenous lipid emulsions (SO-ILE), recognized as a major contributing factor [4], [5]. Strategies to prevent and manage IFALD include modifications in parenteral lipid dosing, such as reducing SO-ILE intake and utilizing alternative lipid formulations, including lipid emulsions containing fish oil [5], [6], [7], [8], [9], [10]. A composite lipid emulsion comprising soybean oil, medium-chain triglycerides, olive oil, and fish oil (SO-ILE, MCT-ILE, OO-ILE, FO-ILE) has been shown to reduce direct bilirubin levels over time; however, its efficacy in reducing the overall incidence of IFALD remains uncertain [8]. In a prior study, SO,MCT,OO,FO-ILE as a therapeutic intervention resulted in IFALD resolution in only 38% of affected patients [5].

Currently, the only lipid emulsion approved by the U.S. Food and Drug Administration for usage in the setting of IFALD in pediatric patients is fish oil-based ILE (FO-ILE) [11]. However, the approved dose of FO-ILE is limited to 1 g/kg/day, thereby restricting the caloric contribution from fat and shifting energy intake toward a higher carbohydrate composition if total daily calorie delivery is maintained [11], [12]. This alteration in macronutrient distribution raises concerns for possible alteration in growth, whether due to decreased total caloric intake or increased carbohydrate intake used to compensate for restricted lipid provision [13]. While FO-ILE has demonstrated comparable growth outcomes to SO-ILE therapy over time [12], [14], only limited data comparing FO-ILE and SO,MCT,OO,FO-ILE exists to our knowledge [15]. The primary goal of this project was to compare growth in neonates with IFALD who received either FO-ILE or SO,MCT,OO,FO-ILE as a treatment strategy. We hypothesized that neonates receiving FO-ILE would have decreased length and increased weight gain relative to those neonates receiving SO,MCT,OO,FO-ILE.

## Material and methods

### Study design and population

This was a retrospective cohort study comparing patients with IFALD receiving SO,MCT,OO,FO-ILE (SMOFlipid, Fresenius Kabi, Uppsala, Sweden) or FO-ILE (Omegaven, Fresenius Kabi, Graz, Austria) in our level IV Neonatal Intensive Care Unit (NICU) [16]**.** The goal of the study was to compare growth outcomes and laboratory data associated with SO,MCT,OO,FO-ILE and FO-ILE therapy in IFALD across two treatment periods. Patients were divided based on the lipid strategy used during their treatment period: the SO,MCT,OO,FO-ILE cohort (January 2016–September 2020) and the FO-ILE cohort (October 2020–May 2024). These time periods were based on lipid availability in the pharmacy formulary and guideline development on lipid use at our institution. Our local practice guideline was developed by our Nutrition Support Team (NST) and includes use of fish oil based ILEs when direct bilirubin is persistently elevated above 2 mg/dL. During the period 2016 – September 2020 our practice included use of SO,MCT,OO,FO-ILE and during October 2020 – 2024 use of FO-ILE. For SO,MCT,OO,FO-ILE doses were started at 1 g/kg/day, titrated daily by 1 g/kg/day up to a goal of 3 g/kg/day. Ordered doses were maintained at 2.5–3 g/kg/day unless enteral feedings were started, in which case lipid doses would be weaned with weaning overall PN components. We did not practice lipid minimization with SO,MCT,OO,FO-ILE in our NICU. Other differences between these two time periods were minimal. However, of note, during the FO-ILE treatment period SO,MCT,OO,FO-ILE was occasionally administered prior to the development of IFALD in high-risk infants. During both time periods, the most common baseline ILE prior to study ILE was SO-ILE. Cohort assignment and data collected was based on ILE used for IFALD treatment. During both time periods infants requiring alternate ILE dosing were co-managed by the NST and primary neonatology team. The NST made suggestions regarding PN and ILE dosing and enteral nutrition advancement with the neonatal team making final decisions. As part of local practice during both time periods essential fatty acid panels were obtained on infants on non-SO-ILE approximately monthly pending risk factors and prior levels.

Neonates with IFALD were eligible if treated January 2016 – September 2020 with SO,MCT,OO,FO-ILE or October 2020 – May 2024 with FO-ILE. IFALD was defined as a direct bilirubin > 2 mg/dL after receiving > 2 weeks of PN. Exclusion criteria included patients with direct bilirubin elevation > 2 mg/dL before two weeks of age, congenitally acquired infections, liver disease unrelated to IFALD, metabolic conditions, and/or genetic diagnoses that could limit growth trajectory. We also later excluded infants who received study ILE for less than seven days. This decision was made as growth data, specifically head circumference (occipital frontal circumference (OFC)) and length, were recorded once weekly per unit protocol.

### Data collection

Data was collected through chart review via the electronic medical record and stored securely in the Research Electronic Data Capture (REDCap) platform provided by our institution [17], [18]. Basic patient demographics were noted, including birth gestational age and underlying diagnosis. To better compare groups, ostomy location and surgery count were recorded, as well as duration of study ILE. The primary outcome was the growth differences of neonates with IFALD while receiving either SO,MCT,OO,FO-ILE or FO-ILE, analyzing the rate of change in weight, length, and OFC z-score from initiation until the completion of the study ILE. The 2013 Fenton growth charts were used for z-score calculation until 52 weeks corrected gestational age and the World Health Organization growth charts were used after this time period if necessary [19]. Growth velocity was also calculated as the change in weight, length, or OFC between end of study ILE and start divided by the number of days of study ILE.

Secondary objectives included describing and comparing the rates of various outcomes between neonates receiving each ILE. We documented the rate of intestinal failure diagnosis in each group, defined as the need for PN for a minimum of 60 days in a 74-day period [2]. While IFALD was a required diagnosis to be included in the study, IFALD can be diagnosed as early as two weeks into the PN course. For this reason, the rate of true intestinal failure could vary between groups and this rate was noted. Additional outcomes included the direct and total bilirubin levels and liver function test changes over time. We also documented nutritional intake including enteral feeding and parenteral intake on a weekly basis, noting calories, protein, carbohydrate, and lipid doses received and averaging over time. To determine if fluid balance influenced the differences in growth, we also recorded the daily intake volumes, including both enteral and parenteral sources. Additional outcomes documented included the rate of infection, mortality, and essential fatty acid panel results. Infection was defined as a positive blood, urine, or cerebrospinal fluid (CSF) culture obtained during hospitalization. Essential fatty acid deficiency (EFAD) was defined as a triene:tetraene (T:T) ratio > 0.2. All data was collected only during the initial hospital stay.

### Statistical analysis

The study was reviewed by the local institutional review board and considered exempt given its retrospective nature (IRB #23359). All patients meeting inclusion criteria during the specified time periods were included in analysis, with the exception of those who received study ILE less than seven days as noted. Continuous variables are presented as median and interquartile range (IQR) and were compared using Mann-Whitney test or two-sided independent *t*-test dependent on distribution of data**.** Data distribution normality was determined using the Shapiro-Wilk test with a p-value of > 0.05 used as the threshold. Categorical variables are presented as number of individuals and percentage and were compared using Fisher’s Exact Test. To further assess the influence of various factors on the primary outcome of growth, multiple linear regression was performed. A two-sided p-value of < 0.05 was used to define statistical significance for all analyses. All statistical analyses were performed using GraphPad Prism version 10.4.1 (GraphPad Software, Boston, MA, USA, graphpad.com).

## Results

**Fig. 1:**
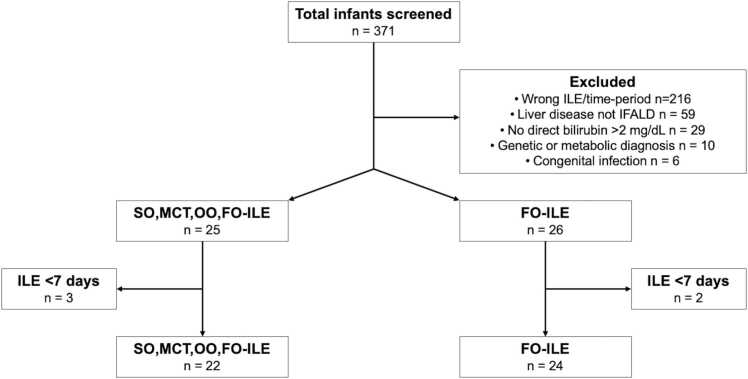
Flow diagram showing patients screened and final inclusion totals. FO-ILE: fish-oil intravenous lipid emulsion; IFALD: intestinal failure associated liver disease; ILE: intravenous lipid emulsion; SO,MCT,OO,FO-ILE: soybean, medium chain triglyceride, olive, fish-oil intravenous lipid emulsion.

An initial 51 patients were identified from the overall population (25 SO,MCT,OO,FO-ILE and 26 FO-ILE), however five patients received study ILE less than seven days (Fig. 1). A total of 46 patients were included in statistical analysis, 22 receiving SO,MCT,OO,FO-ILE and 24 FO-ILE. Table 1 notes the demographic and baseline characteristics of the two ILE groups. The groups had similar gestational ages, birth weights, and underlying diagnoses. Of note, the FO-ILE infants were older with a greater corrected gestational age at study ILE initiation. More of the patients receiving FO-ILE met the diagnosis of intestinal failure (SO,MCT,OO,FO-ILE 55% versus FO-ILE 96%; p = 0.001). The location of patient ostomies and number of surgeries during the hospitalization also varied by lipid group.

**Table 1:** Demographic and population comparisons by lipid treatment group. (All data presented as median (IQR) unless otherwise specified.)

**Parameter**		**SO,MCT,OO,FO-ILE (n = 22)**	**FO-ILE** **(n = 24)**	**p-value**
Birth Gestational Age (weeks)		26.7 (25.0, 28.6)	26.9 (25.2, 33.0)	0.475^†^
Birth weight (kg)		0.788 (0.567, 1.018)	0.906 (0.693, 1.933)	0.064^†^
Age at starting study ILE (days)		46 (33.8, 73.5)	66 (49.5, 89)	0.037^‡^
Corrected Gestational Age at starting ILE (weeks)		34.7 (31.7, 37.9)	38 (34.3, 41.4)	0.016^†^
Diagnosis n (%)	Necrotizing enterocolitis	10 (45)	13 (54)	0.284^§^
	Gastroschisis	1 (4.5)	6 (25)	
	Intestinal atresia	3 (14)	3 (13)	
	Intestinal obstruction	2 (9)	1 (4)	
	Volvulus	1 (4.5)	0	
	Spontaneous intestinal perforation	1 (4.5)	0	
	Imperforate anus	1 (4.5)	0	
	Other	3 (14)	1 (4)	
Ostomy present n (%)		15 (68)	18 (75)	0.746^§^
Ostomy location n (%)	Duodenostomy	1 (7)	2 (11)	0.035^§^
	Jejunostomy	2 (13)	9 (50)	
	Ileostomy	9 (60)	7 (39)	
	Colostomy	3 (20)	0	
Intestinal failure diagnosis n (%)		12 (55)	23 (96)	0.001
Number of abdominal surgeries		2 (1, 2)	2 (2, 3)	0.027^†^
Baseline direct bilirubin (mg/dL)		3.9 (3.4, 4.4)	3.4 (3.0, 4.1)	0.102^†^
Baseline total bilirubin (mg/dL)		6 (5.2, 7.8)	5.3 (4.4, 6.1)	0.028^†^
Total days of study ILE		16.5 (10.8, 21.3)	23.5 (17, 36.5)	0.054^†^

FO-ILE: fish-oil intravenous lipid emulsion; IQR: interquartile range; ILE: intravenous lipid emulsion; SO,MCT,OO,FO-ILE: soybean, medium chain triglyceride, olive, fish-oil intravenous lipid emulsion

†Mann-Whitney used for analysis; ^‡^*t*-test used for analysis; ^§^Fisher’s text used for analysis.

**Fig. 2:**
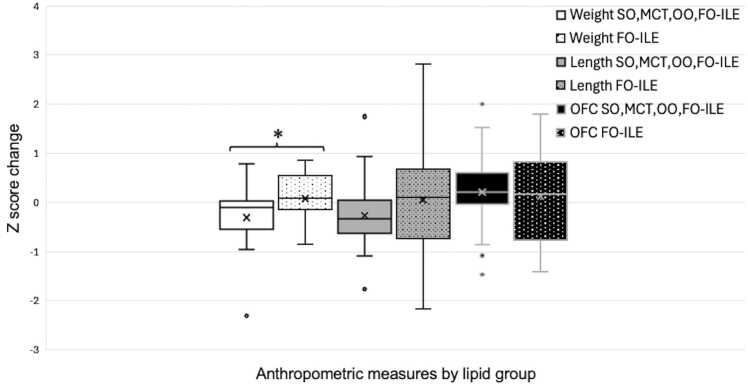
Z-score change over time compared between lipid treatment groups. Only the comparison between weight z-score change was significant SO,MCT,OO,FO-ILE −0.11 (IQR −0.54, 0.04) versus FO-ILE 0.09 (IQR −0.16, 0.54); p = 0.032.^†^ Length: SO,MCT,OO,FO-ILE −0.33 (IQR −0.63, 0.06) versus FO-ILE 0.10 (IQR −0.75, 0.68); p = 0.382.^‡^ OFC: SO,MCT,OO,FO-ILE 0.21 (IQR −0.04, 0.61) versus FO-ILE 0.17 (IQR −0.78, 0.83); p = 0.718.^‡^. * p-value < 0.05; ^†^Mann-Whitney used for analysis; ^‡^*t*-test used for analysis.

Growth data is presented in Fig. 2. When comparing change in z-score, there was no significant difference in the rate of OFC or length growth between the treatment groups. There was a significant difference, however, in the rate of weight z-score change between groups with the SO,MCT,OO,FO-ILE group having a decrease in weight z-score over time compared to an increase in weight z-score over time in the FO-ILE group (SO,MCT,OO,FO-ILE −0.11 (IQR −0.54, 0.04) versus FO-ILE 0.09 (IQR −0.16, 0.54); p = 0.032). When assessing the difference as weight gain velocity, there was no statistically significant difference between groups (SO,MCT,OO,FO-ILE 24.6 g/day (IQR 20.3, 35.3) versus FO-ILE 31.6 g/day (IQR 22.7, 40.8); p = 0.121). There was also no difference in length velocity (SO,MCT,OO,FO-ILE 0.15 cm/day (IQR 0.09, 0.21) versus FO-ILE 0.11 cm/day (IQR 0, 0.18); p = 0.146) or OFC velocity (SO,MCT,OO,FO-ILE 0.13 cm/day (IQR 0.08, 0.20) versus FO-ILE 0.10 cm/day (IQR 0.04, 0.14); p = 0.054) over time.

Nutritional intake, including enteral and parenteral, is described in Table 2. SO,MCT,OO,FO-ILE patients received greater total calories than FO-ILE patients (112 kcal/kg/day (IQR 104.6, 117.1) versus 100.7 kcal/kg/day (IQR 91.0, 109.9); p = 0.004). This higher intake included a greater ILE dose (SO,MCT,OO,FO-ILE 2.1 g/kg/day (IQR 1.8, 2.4) versus FO-ILE 1.0 g/kg/day (IQR 0.9, 1.2); p < 0.001) and enteral calories (SO,MCT,OO,FO-ILE 30.6 kcal/kg/day (IQR 21.2, 38.0) versus FO-ILE 21.4 kcal/kg/day (IQR 13.4, 27.6); p = 0.023). The FO-ILE group, however, received a higher carbohydrate dose with a higher glucose infusion rate (SO,MCT,OO,FO-ILE 9.6 mg/kg/min (IQR 7.1, 10.8) versus FO-ILE 12.8 (IQR 10.0, 13.4); p < 0.001). There was no difference in amino acid dose, total parenteral nutrition calories, or total daily intake volume.

**Table 2:** Nutrition intake by lipid group presented as average daily intake received. (All data presented as median (IQR) unless otherwise specified.)

**Parameter**	**SO,MCT,OO,FO-ILE (n = 22)**	**FO-ILE** **(n = 24)**	**p-value**
Total calories (kcal/kg/d)	112.0 (104.6, 117.1)	100.7 (91.0, 109.9)	0.004^‡^
Enteral calories (kcal/kg/d)	30.6 (21.2, 38.0)	21.4 (13.4, 27.6)	0.023^†^
PN calories (kcal/kg/d)	82.3 (69.4, 93.5)	86.2 (73.8, 93.8)	0.776^‡^
ILE dose (g/kg/d)	2.1 (1.8, 2.4)	1.0 (0.9, 1.2)	< 0.001^‡^
Amino acid dose (g/kg/d)	3.1 (2.4, 3.3)	3.3 (3.0, 3.5)	0.066^†^
Glucose infusion rate (mg/kg/min)	9.6 (7.1, 10.8)	12.8 (10.0, 13.4)	< 0.001^†^
Total volume intake (mL/kg/day)	135.9 (130.4, 150.5)	132.1 (123.7, 145.9)	0.197^‡^

FO-ILE: fish-oil intravenous lipid emulsion; IQR: interquartile range; ILE: intravenous lipid emulsion; PN: parenteral nutrition; SO,MCT,OO,FO-ILE: soybean, medium chain triglyceride, olive, fish-oil intravenous lipid emulsion

†Mann-Whitney used for analysis; ^‡^*t*-test used for analysis.

Secondary outcomes are shown in Table 3. Laboratory outcomes are presented as the difference in change of laboratory parameter over time. The FO-ILE group showed a decline in total bilirubin and direct bilirubin over time while the SO,MCT,OO,FO-ILE group showed an increase in these values. About a third of patients in each group had essential fatty acid panels obtained (SO,MCT,OO,FO-ILE 6/22 (27%); FO-ILE (7/24 (29%)). The results of these panels are presented in Table 3. Those in the SO,MCT,OO,FO-ILE group had higher levels of linoleic acid but lower levels of docosahexaenoic acid and eicosapentaenoic acid. Only one patient had EFAD with a T:T ratio of 0.212 in the SO,MCT,OO,FO-ILE group six weeks into therapy. At the time of the panel, they were receiving an average of 1.6 g/kg/day of SO,MCT,OO,FO-ILE with dose increased to an average of 2.3 g/kg/day repeat T:T ratio five weeks later was 0.019. There was no difference between the groups when comparing the rate of infections or mortality.

**Table 3:** Patient outcomes by lipid treatment group. (All data presented as median (IQR) unless otherwise specified.)

**Parameter**	**SO,MCT,OO,FO-ILE (n = 22)**	**FO-ILE** **(n = 24)**	**p-value**
Triglycerides while on ILE (mg/dL)	97.8 (63.6, 135.1)	101 (76, 124)	0.838^‡^
Change in AST while on ILE (Units/L/d)	0.7 (−1.3, 4.5)	−0.7 (−2.1, 0.7)	0.077^†^
Change in ALT (Units/L/d)	1.4 (−0.3, 4.1)	0.2 (−3.0, 1.4)	0.096^‡^
Change in total bilirubin on ILE (mg/dL/d)	0.09 (−0.05, 0.18)	−0.04 (−0.09, 0.03)	0.013^‡^
Change in direct bilirubin on ILE (mg/dL/d)	0.05 (−0.04, 0.10)	−0.04 (−0.08, 0.01)	0.024^‡^
Essential Fatty Acid Panel Results Number of patients (%) Linoleic Acid (nmol/mL) α-linolenic Acid (nmol/mL) Arachidonic Acid (nmol/mL) DHA (nmol/mL) EPA (nmol/mL) Mead Acid (nmol/mL) Triene:tetraene ratio	6 (27) 1347 (800.3, 2007) 61.5 (28.5, 133.8) 26 (23.3, 274) 238 (169.8, 272.5) 166 (123.5, 224.8) 25 (16, 37) 0.074 (0.049, 0.166)	7 (29) 676 (513, 809) 41 (24, 60) 305 (26, 383) 890 (628, 1123) 678 (618, 1188) 14 (6, 22) 0.033 (0.016, 0.057)	0.010^‡^ 0.158^‡^ 0.100^†^ 0.002^‡^ 0.003^‡^ 0.061^‡^ 0.050^‡^
Rate of infection n (%)	15 (68)	12 (50)	0.245^§^
Type of positive culture			0.239^§^
Blood	7	9	
Urine	7	3	
CSF	0	0	
Blood and urine	1	0	
Mortality	2 (9)	1 (4)	0.600^§^

DHA: Docosahexaenoic acid; EPA: Eicosapentaenoic acid; FO-ILE: fish-oil intravenous lipid emulsion; IQR: interquartile range; ILE: intravenous lipid emulsion; PN: parenteral nutrition; SO,MCT,OO,FO-ILE: soy oil, medium chain triglyceride, olive oil, fish oil intravenous lipid emulsion

†Mann-Whitney used for analysis; ^‡^*t*-test used for analysis; ^§^ Fisher’s text used for analysis.

To determine variables associated with growth differences, multiple linear regression was performed for both z-score change and weight velocity. For both analyses, the variables included in analysis were those that varied significantly between groups or were deemed likely to influence the rate of weight gain including: ILE dose, birth weight, age at starting study ILE, ostomy location, intestinal failure diagnosis, days of lipid therapy, enteral calories, glucose infusion rate, and change in direct bilirubin. In multiple linear regression analyses for change in weight z-score variable selection, only ILE dose was retained in the model (p = 0.016 with R^2^ 0.448). When weight velocity was also assessed in multiple linear regression using the same variables, no variables were retained in the model as all were non-significant with p-value > 0.05.

## Discussion

In this study, neonates with IFALD receiving either SO,MCT,OO,FO-ILE or FO-ILE demonstrated comparable growth velocity during treatment. To our knowledge, no direct growth comparisons between patients receiving SO,MCT,OO,FO-ILE or FO-ILE in the setting of IFALD have been conducted previously. Ramiro-Cortijo et al. compared outcomes for patients who had previously received SO,MCT,OO,FO-ILE and were transitioned to FO-ILE due to worsened cholestasis [15]. They reported growth during each of these treatment time periods; however they did not directly compare the findings. In the setting of SO-ILE compared to FO-ILE Gura et al. noted similar weight, length, and OFC z-scores between groups receiving SO-ILE or FO-ILE at time of cholestasis resolution and end of the study [12]. In this study, similar to our population, those receiving FO-ILE received significantly less calories from ILE and more from parenteral glucose. The finding of similar weight gain velocity between groups is clinically important as higher weight gain velocity during initial hospitalization has been associated with improved neonatal outcomes, including improved later neurodevelopment [20].

While weight gain velocity was similar, we did note a significant increase in weight z-score over time in the FO-ILE group compared to those receiving SO,MCT,OO,FO-ILE, despite the latter group receiving a higher total caloric intake and greater enteral nutrition. This significant finding in association with a lack of significant difference in growth velocity between groups may be explained by variations in age and corrected gestational age at the time of study lipid initiation. As growth velocity naturally varies with infant age, z-score change offers a more appropriate metric for comparing growth across groups of differing ages. Given our FO-ILE group was older and of a greater corrected gestational age at the time of study ILE initiation, they would be expected to gain less weight over time to maintain or improve their weight z-score. Others have noted increased weight gain with FO-ILE; however, this was in comparison to SO-ILE reduced dosing with similar dosing of all PN components including ILE [21].

In our population, the FO-ILE group received lower ILE dosing and increased glucose supplementation. It is well known that excess glucose beyond oxidative and glycogen storage capacities is converted to lipids through lipogenesis, potentially contributing to increased fat deposition [13]. In our study, while the weight gain velocity was maintained, the disproportionate weight z-score relative to length observed in the FO-ILE group raises concern for altered body composition. Weight-for-length has been assessed for correlation with body composition, specifically fat mass, in multiple studies, although the findings from these studies are inconsistent [22], [23]. The retrospective nature of our current study, with no documentation of body composition, does not allow us to draw conclusions regarding body composition of our population. However, questions and concerns are raised regarding this growth differences between groups, highlighting the need for further studies to analyze the body composition in this population and the setting of variable ILE and PN dosing strategies.

Our study further demonstrated that neonates receiving FO-ILE exhibited a greater reduction in direct bilirubin and total bilirubin levels over time compared to those receiving SO,MCT,OO,FO-ILE. These findings align with previous studies, which have reported significant decreases in bilirubin levels among cholestatic neonates transitioned from SO,MCT,OO,FO-ILE to FO-ILE [15], [24]. This effect is consistent with the known hepatoprotective properties of FO-ILE, which are thought to mitigate IFALD through anti-inflammatory mechanisms, including increased omega−3 fatty acid content and minimal phytosterol content [25]. Furthermore, multiple studies support these findings, as FO-ILE-treated patients demonstrated a high rate of cholestasis resolution, ranging from 50% to 86% achieving direct bilirubin levels below 2 mg/dL [10], [26]. These results highlight the role of FO-ILE in the management of IFALD, suggesting that its incorporation into neonatal parenteral nutrition regimens may improve hepatic outcomes in this vulnerable population.

Only a fraction of our study population had essential fatty acid levels obtained. Comparisons of fatty acid profiles between groups showed slight differences, which may primarily reflect the inherent fatty acid composition of the ILEs themselves, as previously described in the literature [27]. It is important to note in this population one patient did have EFAD as evidenced by a T:T ratio > 0.2 while receiving a SO,MCT,OO,FO-ILE dose lower than our local recommended dosing of 2.5–3 g/kg/day. EFAD in the setting of restrictive doses of SO,MCT,OO,FO-ILE has been described in neonates [28], [29]. While our local practice is to order SO,MCT,OO,FO-ILE at 2.5–3 g/kg/day it is important to consider what the neonate is receiving, which is what we documented in this study. With compatibility concerns it is not uncommon for infants to receive lower doses of SO,MCT,OO,FO-ILE than intended, the likely cause in our patient with EFAD. In clinical practice it is important to monitor the SO,MCT,OO,FO-ILE dose received and note reasons for stopping the infusion. In comparison, FO-ILE monotherapy has not been associated with EFAD in the literature and has actually been used to treat EFAD in patients who cannot tolerate other ILE sources in case reports [30], [31]. In our small cohort of patients with fatty acid levels measured, no FO-ILE patients had EFAD.

In addition to growth and laboratory outcomes we also reported rates of additional complications including infections and mortality. The overall rate of mortality in our population was low and did not differ between treatment groups. Additionally, there was no difference between groups with regards to the rate of infection, or the type of infection in those with positive cultures. This finding is notable, as prior studies have reported infants with inflammatory morbidities, such as infection, may experience impaired growth compared with those without [12]. Our local clinical practice does not include regular monitoring of inflammatory markers outside of times when concerns for infection arise. Because of this, we could not include other inflammatory markers, such as C-reactive protein or procalcitonin, as other markers of inflammation that could correlate with growth outcomes [32].

We assessed patient growth only while receiving the study lipid emulsion and did not evaluate growth trajectories before starting or after discontinuation of lipid therapy or long-term outcomes. The retrospective nature also limits the ability to fully draw conclusions or causality from this study alone. Our regression model notes that only 45% of the variation in patient weight z-score is accounted for by the ILE dose. While the two groups were from two different treatment time periods, they were treated at a single institution by the same group of providers using similar protocols aside from ILE formulation and dosing. We attempted to assess other factors that could influence weight gain trajectory, such as average daily fluid intake, which did not vary between groups. However, despite these similarities, unmeasured differences in patient characteristics or practice changes over time may have influenced growth outcomes beyond the parameters captured in this study.

## Conclusion

To our knowledge, this is the first study to compare growth outcomes between neonates with IFALD receiving SO,MCT,OO,FO-ILE or FO-ILE. Infants treated with FO-ILE maintained similar weight gain velocity while demonstrating reductions in direct bilirubin during therapy, suggesting that FO-ILE may support adequate growth while improving cholestasis. Although we observed an increase in weight z-score among patients receiving FO-ILE compared to a decrease in weight z-score in those with SO,MCT,OO,FO-ILE, we cannot draw conclusions from this study alone about the clinical relevance of this finding. Future studies that include body composition analysis are needed to truly understand this finding. Additionally, multicenter studies with a larger patient population would help to better understand both the short and long-term impact of these lipid strategies on important neonatal outcomes including neurodevelopment.

## Ethical Clearance

Ethics approval was obtained and deemed exempt from review with the letter dated May 31, 2024, under IRB #233359. All research was carried out in accordance with the Declaration of Helsinki and all procedures were in compliance with relevant laws and guidelines.

## Funding

This research did not receive any specific grant from funding agencies in the public, commercial, or not-for-profit sectors.

## CRediT authorship contribution statement

**Huff Katie:** Writing – review & editing, Supervision, Software, Project administration, Methodology, Investigation, Formal analysis, Conceptualization. **Emily A. Gutzwiller:** Writing – original draft, Methodology, Investigation, Data curation.

## Declaration of Competing Interest

The authors declare the following financial interests/personal relationships which may be considered as potential competing interests. Katie Huff reports a relationship with Baxter Healthcare that includes: speaking and lecture fees. Katie Huff reports a relationship with Alcresta Therapeutics Inc that includes: consulting or advisory. If there are other authors, they declare that they have no known competing financial interests or personal relationships that could have appeared to influence the work reported in this paper.
